# Supplementary material for: Identification of photoperiod-induced specific miRNAs in the adrenal glands of Sunite sheep (Ovis aries)
Source: Front Vet Sci. 2022 Jul 22;9:888207. doi: 10.3389/fvets.2022.888207 (PMC9354845; doi:10.3389/fvets.2022.888207)
Supplement: Supplementary file 1 [file Data_Sheet_1.ZIP › Supplementary file/miRDeep2 file.zip!/miRDeep2 file/Know_Structure/SPLP42A3/oar-mir-194.pdf]

miRBase precursor : oar-mir-194
Total read count : 3316
oar-miR-194 read count : 3314
remaining reads : 2

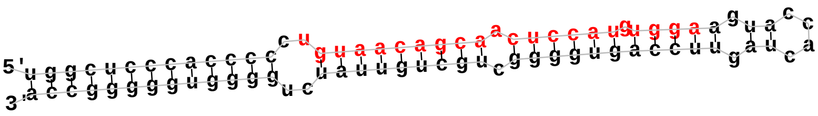

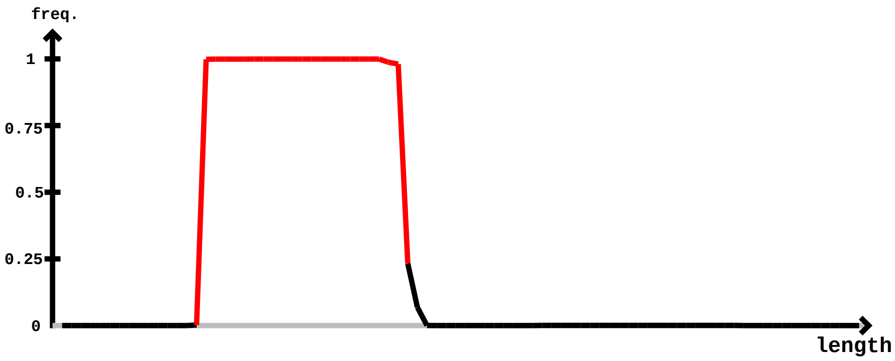


```
                  oar-miR-194
5'- uggcucccacccccuguaacagcaacuccaugugga aguaccacuaguuccaguggggcugcuguuaucuggggugggggcca  -3'   exp
    (((((((((((((..(((((((((.((((((.(((((.((....)).))))))))))).)))))))))..)))))))))))))    reads    mm    sample
    .............cuguaacagcaacuccaugu..................................................    1        0     seq
    .............cuguaacagcaacuccaugugg................................................    5        0     seq
    .............cuguaacagcaacuccaugugga...............................................    1        0     seq
    ..............uguaacagcaacuccaugu..................................................    40       0     seq
    ..............uguaacagcaacuccaugug.................................................    21       0     seq
    ..............uguaacagcaacuccaugugg................................................    2475     0     seq
    ..............uguaacagcaacuccaugugga...............................................    543      0     seq
    ..............uguaacagcaacuccauguggaC..............................................    11       1     seq
    ..............uguaacagcaacuccauguggaa..............................................    213      0     seq
    ..............uguaacagcaacuccauguggaag.............................................    1        0     seq
    ...............guaacagcaacuccaugugg................................................    2        0     seq
    ...................cagcaacuccauguggaa..............................................    1        0     seq
    .................................................ccaguggggcugcuguuaucu.............    2        0     seq
```
